# School Health Promotion, the Body Mass Index z-Score, and Psychosocial Health in Primary Schools of the Netherlands

**DOI:** 10.3390/ijerph21081073

**Published:** 2024-08-15

**Authors:** Lisanne Vonk, Iris Eekhout, Tim Huijts, Mark Levels, Maria Jansen

**Affiliations:** 1 Department of Health Services Research, Care and Public Health Research Institute (CAPHRI), Maastricht University, P.O. Box 616, 6200 MD Maastricht, The Netherlands; maria.jansen@maastrichtuniversity.nl; 2 Academic Collaborative Center for Public Health Limburg, Public Health Service South Limburg, P.O. Box 33, 6400 AA Heerlen, The Netherlands; 3 Expertise Center Child Health, Netherlands Organisation for Applied Scientific Research (TNO), P.O. Box 3005, 2301 DA Leiden, The Netherlands; iris.eekhout@tno.nl; 4 Research Centre for Education and the Labour Market (ROA), School of Business and Economics, Maastricht University, P.O. Box 616, 6200 MD Maastricht, The Netherlands; t.huijts@maastrichtuniversity.nl (T.H.); m.levels@maastrichtuniversity.nl (M.L.)

**Keywords:** school health promotion, multilevel analysis, primary school, BMI z, SDQ

## Abstract

Childhood overweight and psychosocial issues remain significant public health concerns. Schools worldwide implement health promotion programs to address these issues and to support the physical and psychosocial health of children. However, more insight is needed into the relation between these health-promoting programs and the Body Mass Index (BMI) z-score and psychosocial health of children, while taking into account how school factors might influence this relation. Therefore, we examined whether the variation between primary schools regarding the BMI z-score and psychosocial health of students could be explained by school health promotion, operationalized as Healthy School (HS) certification, general school characteristics, and the school population; we also examined to what extent the characteristics interact. The current study had a repeated cross-sectional design. Multilevel analyses were performed to calculate the variation between schools, and to examine the association between HS certification and our outcomes. Existing data of multiple school years on 1698 schools were used for the BMI z-score and on 841 schools for psychosocial health. The school level explained 2.41% of the variation in the BMI z-score and 2.45% of the variation in psychosocial health, and differences were mostly explained by parental socioeconomic status. Additionally, HS certification was associated with slightly lower BMI z-scores, but not with psychosocial health. Therefore, obtaining HS certification might contribute to the better physical health of primary school students in general. This might indicate that HS certification also relates to healthier lifestyles in primary schools, but further research should examine this.

## 1. Introduction

Childhood overweight continues to be a severe public health concern in the Western world, as a recent report from the World Health Organization (WHO) on 33 countries in the European Region showed that 29% of 7–9-year-old children were overweight or obese [1]. This is problematic, since overweight can lead to serious physical health issues later in life, such as cancer and hypertension [2]. Being overweight is also related to a worse psychosocial health [3,4], defined by Vannieuwenborg et al. [5] as “All complaints which are not strictly medical or somatic. They affect the patient’s functioning in daily life, his or her environment and/or life events. (…)”, and show much overlap with mental health, according to the definition of the WHO [6]. Besides overweight, underweight can also indicate psychosocial health problems [4]. Concerns about psychosocial health have risen for all children, particularly since the start of the COVID-19 pandemic [7], with many recent studies reporting a decline in children’s psychosocial health [8,9,10]. These findings highlight the importance of supporting both physical and psychosocial health outcomes in children, especially since early childhood is a crucial period for the further course of life [11].

To promote the physical and psychosocial health of children, schools worldwide implement health-promoting programs [12]. Despite their widespread implementation, Langford et al. [12] identified some knowledge gaps and methodological issues in the existing literature. Research on psychosocial health in the context of school health promotion (SHP) is notably sparse [12,13]. For physical health, most studies focusing on weight status included Body Mass Index (BMI) as an outcome, but the authors concluded that the BMI z-score should be used instead [12], since this allows for more accurate comparisons between students of varying ages and genders. Additionally, their review included studies that evaluated SHP and focused on BMI or BMI z as an outcome, but findings were inconsistent [12]. A possible explanation is that schools function as complex adaptive systems, meaning that the implementation of a health-promoting program could induce different responses, depending on the specific context of each school [14]. A study by Bartelink et al. [15] also showed that a health-promoting program focusing on physical activity, as well as dietary intake in some schools, was effective in decreasing the BMI z-scores of primary school students, but different effects were observed between schools [16]. Nevertheless, these results were based on data from eight primary schools from one small region in the Netherlands with a relatively high level of social deprivation, which means that the external validity of these findings is difficult to assess.

Given these knowledge gaps, it is important to gain a deeper understanding of the relation between SHP and the BMI z-score and psychosocial health. Additionally, it is important to enhance our comprehension of how SHP interacts with the school context. Our study was conducted in the Netherlands and evaluated the Healthy School (HS) program, which employs a whole-school approach. The following questions were answered: (a) ‘To what extent can the variation between primary schools in the Netherlands regarding the BMI z-score and psychosocial health of students be explained by differences regarding SHP, operationalized by HS certification, general school characteristics, and school population characteristics?’ and (b) ‘To what extent is SHP associated with the BMI z-score and psychosocial health of primary school students, and is this association moderated by general school characteristics and school population characteristics?’ Given the relationship between weight status and psychosocial health [3,4,17], it is assumed that enhancing physical health can positively impact psychosocial health and vice versa. Therefore, we hypothesized that HS certification is related to the BMI z-score and psychosocial health of students, and thus may contribute to health overall.

## 2. Materials and Methods

### 2.1. Study Design and Study Population

The design of the current study was a repeated cross-sectional multilevel design. In the Netherlands, the Youth Health Care (YHC) invites every child regularly for routine contact moments to monitor their health and development. For this study, anonymous data from two consecutive contact moments were used—at or around the age of 5/6 and at the age of 9/10/11. Data were obtained from the Netherlands Cohort Study on Education (NCO) regarding general school characteristics and other school population characteristics, and data on school health promotion were used from the HS organization. Most characteristics varied per school year.

Data were collected of primary school students from the assessment at age 5/6 and age 9/10/11 from seven out of the twenty-five Public Health Services (PHSs). Three schools explicitly stated that their school’s data could not be used and the data of their students were therefore not obtained for this study. Digitalized data from students who were assessed by the YHC in the school years 2014–2015 up to and including 2018–2019 were included. For the assessment at age 5/6, students born between 2008 and 2014 were included. For the assessment at age 9/10/11, students born between 2003 and 2009 were included. Students without a school identifier, from special needs schools, or with an unknown measurement moment were excluded. If the identifier for the school branch was missing and the school had only one branch, we assumed the data belonged to that school. If a student appeared multiple times in the dataset at one contact moment (0.2%), e.g., by visiting the PHS more often, only one visit was included. Students from schools that were not identified in the NCO dataset were also excluded, as well as students without any data with regard to our outcomes (weight, height, and the Strengths and Difficulties Questionnaire score (SDQ)). For our analyses concerning psychosocial health, students from PHSs without information on the separate SDQ items were excluded. In case data were missing for many students or there was a presumed high risk of selection or information bias, the data of the PHS were excluded for that particular school year. This was determined for both contact moments and outcome variables separately. For our analyses concerning the BMI z-score, students with missing data were excluded, since this concerned relatively few students (2.3%). Lastly, schools were excluded if data from fewer than five students were available within one school year. Due to our method for data collection and exclusion criteria, the number of included school years could differ between PHSs. The Ethics Review committee of the faculty of Health, Medicine, and Life Sciences of Maastricht University approved the current study (FHML-REC/2020/083.01).

#### Measurements

*Outcomes.* Our outcomes were the BMI z-score and psychosocial health, which was operationalized as the total SDQ score [18]. To calculate the BMI z-score, an YHC professional conducted height and weight measurements with a scale and a stadiometer or microtoise [19]. National guidelines exist for YHC professionals on measuring weight and height. However, protocols for whether children were weighed with or without clothing could vary across PHSs, age groups, and school years. Whether a student was measured with clothes was based on the PHS’ protocol, unless the measurement method was registered explicitly. According to the guidelines of the Netherlands Center Youth Help, measurements with clothes subtracted 0.4 kg for 4 to 8 year olds, 0.6 kg for 9 to 11 year olds, and 0.8 for 12 year olds [19]. BMI z-scores were calculated using the following formula [20]:

(1) $$BMI\;z-score=\left[{\left(BMI÷M\right)}^{L}-1\right]÷\left(L\times S\right)$$

BMI was calculated as weight/height^2^. The L represents the skewness, M the median, and the S the coefficient of variation. Dutch reference values by age and gender were used to calculate these scores [21], which were interpolated for age in months. To measure psychosocial health, parents filled out the SDQ, usually shortly before the height and weight measurements were taken. This validated and reliable questionnaire [22,23] consists of five subscales, each consisting of five questions [18]. All questions have three answer categories that are scored with a range from 0 to 2. Total scores are composed of four subscales and range from 0 to 40 points [18]. According to the guidelines of the Netherlands Organisation for Applied Scientific Research (TNO), scores higher than 10 are considered borderline or abnormal for 4–11 year old students [24].

*School health promotion.* SHP was operationalized by the implementation of the Dutch HS program, since this study is part of the national evaluation of the program [25]. Within the same project, similar studies have been performed regarding the educational performance of primary and secondary school students, and the dietary intake of secondary school students [26,27,28]. The HS program assists primary, secondary, and secondary vocational schools in promoting healthier habits among students. It focusses on health education, school environments, identifying health problems, and health policy [29]. The following characteristics related to the HS program were included: *HS* (indicating whether a school had the HS program certificate in a specific school year); HS ever (whether a school obtained the HS program certificate at least once since the beginning of the program (2010) [30]); and number of years HS (the cumulative duration in years a school has or had been a certified school since the beginning of the program). Within our study period, schools could obtain a topic certificate for eight different health themes, as follows: nutrition; physical activity; well-being; smoking; alcohol and drug prevention; relationships and sexuality; environment and nature; physical safety; and hygiene, skin and teeth. These topic certificates were included as variables in our main analyses, unless less than 10 schools obtained the topic certificate. Schools that meet the requirements of the HS program for a health theme, i.e., for all four pillars (education, environment, signalizing, and policy), can apply for the topic certificate by completing a self-reported questionnaire [29]. When approved, they receive the HS program certificate as well. A certificate is valid for the school year in which it is obtained, and three school years afterwards. August first was designated as the cut-off date. HS certification served as an indicator of implementation adherence to the four pillars.

*General school characteristics.* The following general school characteristics were included: urbanicity of the school area (low (<1000 addresses/km^2^), medium, and high (≥1500 addresses/km^2^)); the school size (i.e., number of students); the school type (public, independent non-denominational education, Catholic, Protestant, and other); and the responsible PHSs for the area in which the school was located.

*School population characteristics.* The following school population characteristics from the NCO dataset were included: disadvantaged students (i.e., the proportion of students with two lower-educated parents); high parental educational attainment (i.e., the proportion of students with at least one higher-educated parent); household income (the proportion of students with a high household income and the proportion of students with a low household income. The proportion of students with a medium household income was used as a reference); and migration background (i.e., the proportion of students with a first-generation migration background and the proportion of students with a second-generation migration background. The proportion of native students was used as a reference). The analyses will look at the urban characteristics of the students’ home area. This includes finding out how many students live in highly urbanized areas and how many live in areas with low urbanism, using data from the YHC. The proportion of students living in a medium urbanicity area was used as a reference (≥1000 and <1500 addresses/km²). For the analyses related to psychosocial health, the proportion of overweight and underweight students was included. Overweight was classified as a BMI z-score > 1 and underweight as a BMI z-score < −2 [31]. Age in months on the day of the weight and height measurement was included as well. Outliers were removed and school and individual data were merged using encrypted school identifiers. The NCO data regarding primary school students in their final year were used to derive a school-level estimate for the variables related to household income, migration background, and high parental educational attainment. Finally, the school year ranging from 2014 to 2018 was also included, with the number indicating the beginning of the school year.

### 2.2. Statistical Analyses

Multilevel regression analyses were conducted using the R package version 4.2.3 [32]. For analyzing psychosocial health, the mice package was used to perform multiple imputations of missing data [33,34], with 10 imputations and 30 iterations. The total SDQ score was calculated based on the results of twenty separate items during the imputation process. To prevent multi-collinearity issues, variables that are composed of other variables were excluded, e.g., such as the presence of an HS program certificate at schools. The difference in health outcomes between schools was accounted for by including the variance in the SDQ score between schools in the imputation model. To enhance our imputations, the following auxiliary variables, i.e., characteristics that are related to the probability of missing data, were included: students’ gender, the contact moment, and the SDQ items for the subscale pro-social behavior that is not included in the total SDQ score. Imputation was not necessary for the BMI z-score, since students with missing data were excluded.

A three-level model was used for data analysis—students (1), nested in school years (2), nested in schools (3). To answer our first research question, the intra-class coefficient (ICC) was calculated, which can range from 0% to 100%, to examine the amount of variance explained by the school and the school year, using these formulas [35]:

(2) $${ICC}_{school}=\frac{\sigma_{school}^{2}}{\sigma_{school}^{2}+\sigma_{school\;year}^{2}+\varepsilon },\;{ICC}_{school\;year}=\frac{\sigma_{school\;year}^{2}}{\sigma_{school}^{2}+\sigma_{school\;year}^{2}+\varepsilon },$$

where the estimated variance at the school-level is denoted as $\sigma_{school}^{2}$, the estimated variance at the school year level as $\sigma_{school\;year}^{2}$, and the residual variance as ε. The null model was first calculated, including random intercepts for school and school year, to assess the variation in BMI z-scores and psychosocial health. Each variable was then added separately to the null model to determine which ones explained at least 10% of the differences between schools [36,37]. Subsequently, these variables were added together to the model to establish the total amount of variation between schools explained by these characteristics. The same process was conducted at the school year level.

To answer our second research question, the association between the number of years of having the HS program certificate, or the most relevant topic certificates, and the outcomes in schools that obtained the HS program certificate during our study, i.e., schools of which data were available prior to obtaining the HS program certificate and after, was examined. To adjust for confounding factors, we controlled for all variables that accounted for a minimum of 10% of the variation between schools. For the BMI z-score, topic certificates were categorized in the following three groups for each separate school year: (1) the nutrition or physical activity certificate, (2) another topic certificate, (3) no topic certificate. For psychosocial health, the same was carried out, but for the well-being certificate. As a final step, we examined whether associations differed between subgroups, by examining the within-level interactions with either the HS program certificate or the separate topic certificates if significant (*p* < 0.05) differences between topic certificates emerged. To explore this, an interaction term between the significant characteristics in the ICC analyses and the HS certification was included in our model.

Since there might be some differences between PHSs, for example, due to registration, the PHS and standardized age were included in all analyses. Individual characteristic were also included when available, i.e., for the urbanicity of the home area and the proportion of overweight and underweight students. The analysis based on multiple imputation was compared to the complete case analysis.

## 3. Results

### 3.1. Descriptive Statistics

A flowchart is presented in Figure S1 in Supplementary Materials, and descriptive statistics are presented in Table 1 for schools that were a certified school at least once since the initiation of the program (we will refer to these schools as ‘all certified schools’), and for schools that were never a certified school.

We also presented the characteristics of a subsample of certified schools, i.e., schools that obtained the HS program certificate within our study period. When examining the BMI z-score, 1698 primary schools and 278,778 students were included in our ICC analyses. Of these 1698 schools, 210 schools became a certified school during the period of this study. For psychosocial health, 841 primary schools and 127,339 students were included in our ICC analyses. Of these 841 schools, 106 schools became a certified school during the period of our study. The most common topic certificates were physical activity, nutrition, and well-being, which all target our outcomes. Students in all certified schools had significantly higher BMI z-scores compared to students in non-certified schools (−0.14 vs. −0.18). Students in all certified schools had, on average, significantly worse SDQ scores compared to students in non-certified schools (5.80 vs. 5.57). The mean BMI z-score and SDQ score in schools that became a certified school within our study period did not significantly differ from the mean in all certified schools.

### 3.2. Differences in the BMI z-Score

Considering all schools, the ICC regarding the BMI z-score was 2.41% at the school-level when controlling for the PHS and standardized age (Table 2). The ICC decreased by ≥10% for four characteristics, i.e., high parental educational attainment, disadvantaged students, household income, and migration background. The ICC at the school year level was 0.62%, but none of the variables exhibited a decrease of at least 10%.

As a next step, multivariate analyses (Table 3) were performed to adjust for the influence of the school population characteristics that were identified as important (high parental education attainment, disadvantaged students, household income, and migration background) [38], only considering schools that became a certified school in our dataset. When adjusting for these variables, as well as the PHS and standardized age, HS certification seems to matter, whereby students in schools with the HS program certificate had lower BMI z-scores (B = −0.03), which is more favorable. Additionally, students in schools with the nutrition and/or physical activity certificate had significantly lower BMI z-scores compared to students in schools without the HS program certificate (B = −0.04). However, the nutrition and physical activity topic certificate did not significantly differ from other topic certificates regarding the BMI z-score. Additionally, the results presented in Table 4 show that there was a favorable association between the HS program certificate and the BMI z-score (B = −0.06) in schools with students who do not face the noted disadvantages, but this association diminished if the proportion of disadvantaged students increased (B = 0.31), i.e., an increase of 10% means an estimated difference of 0.03 in the BMI z-score. Additionally, household income also moderated the association of the HS program certificate, if 50% of the school population had a high household income instead of a low household income, this leads to an estimated difference of −0.08 in the BMI z-score. Lastly, the HS program certificate had a favorable association with the BMI z-score in schools with only native students (B = −0.04), but this association diminished if the proportion of students with a second-generation migration background increased (B = 0.17), i.e., an increase of 10% means a difference of approximately 0.02 in the BMI z-score.

### 3.3. Differences in Psychosocial Health

Considering all schools, the ICC regarding the SDQ score was 2.45% at the school-level when controlling for the PHS and standardized age (Table 2). The ICC at the school-level decreased by ≥10% for five characteristics—disadvantaged students, high parental educational attainment, household income, migration background, and overweight and underweight students. The ICC at the school year level was 0.45%, but none of the variables exhibited a decrease of at least 10%.

We did not find evidence for an association between HS certification and psychosocial health when considering schools that became a certified school in our dataset (Table 3). There was also no interaction between the HS program certificate and the school population (Table 4). The complete case analyses led to similar conclusions as the analyses based on multiple imputation.

## 4. Discussion

The aim of this study was to examine to what extent differences in BMI z-scores and psychosocial health between primary school students in the Netherlands could be explained by differences between schools regarding school health promotion, operationalized by HS certification, general school characteristics, and school population characteristics. The association between SHP and the outcomes was also examined, and whether this association was moderated by these general school characteristics and the school population. When considering all schools, our findings revealed that 2.41% of the total variation in BMI z-scores and 2.45% of the total variation in psychosocial health could be attributed to disparities between schools. Differences within schools over time accounted for 0.62% and 0.45% of the total variation, respectively.

This indicates that differences in BMI z and psychosocial health are largely explained by individual student characteristics, but that a small part of the BMI z-score and psychosocial health can be attributed to the school-level. For the BMI z-score, most variance between schools was explained by indicators of socioeconomic status (SES), i.e., high parental educational attainment, followed by disadvantaged students and the household income. These results are consistent with the results of previous studies that showed that the SES and weight status of children are related in high-income countries [39]. Moreover, SES indicators are also related to dietary intake and physical activity [40,41,42,43,44,45,46], which are, in turn, determinants of weight status [47]. Families with low household incomes might not be able to afford healthy products such as fruit and vegetables, or playing sports at a sport club [43,48,49,50,51]. Besides SES, migration background also explained differences between schools, which was also in line with previous studies [52]. For psychosocial health, differences between schools were also partly explained by SES indicators and migration background, which was in line with the previous literature [53,54]. Immigrant students might experience more psychosocial health problems due to difficulties with adjusting to new settings, language barriers, and potential adverse experiences [54,55]. Besides SES and migration background, overweight and underweight students also explained the differences between schools in psychosocial health. Another study on adolescents reported that overweight was related to reduced psychosocial health, partly due to being bullied [56]. Furthermore, none of the characteristics substantively explained differences in the BMI z-score and psychosocial health within schools over time.

HS certification did not explain differences between schools or within schools over time for both outcomes. However, for the schools that transitioned to being a certified school within our study period, a small but significant association was observed between having the HS program certificate and students’ BMI z-score when adjusting for the school population characteristics that explained differences between schools, as well as the standardized age and the PHS. We also found a significant association for the nutrition and physical activity certificates compared to not having the HS program certificate, but no significant difference was found compared to the other topic certificates. Since HS certification has a favorable association with the BMI z-score and most topic certificates focus on health behaviors, these might also be related to lifestyle, such as a healthier dietary intake. Additionally, the HS program had a stronger favorable association with the BMI z-score in schools with no disadvantaged students, schools with students with high household incomes, and schools with native students compared to school with students with a second-generation migration background. The favorable association with the HS program in schools with a higher SES school population might be due to the home situation. Bartelink et al. [57] demonstrated that SES moderated the effects of an SHP intervention at home, and results indicated that physical activity and dietary intake at home was less favorable for students with a lower SES. High SES parents may possess greater resources for fostering healthier habits. Another study found that in general, higher-educated mothers had more knowledge and a more positive attitude towards healthy dietary intake compared to lower-educated mothers [58]. These differences might moderate the impact of SHP. For psychosocial health, no association with HS certification was identified, contradicting our hypothesis.

### Strengths and Limitations

Using existing datasets facilitated the inclusion of data from many schools from multiple school years. This enabled us to contribute to the existing literature on SHP. Additionally, weight and height were measured by professionals instead of being self-reported. This decreased the chance of information bias. This study employed a repeated cross-sectional multilevel design, allowing for the measurement of changes within schools over time.

However, since our study depended on existing databases, this also provided limitations that should be taken into account when interpreting the results. With regard to our outcomes, not all potentially important characteristics could be incorporated, such as characteristics of the home environment [57,59,60], as well as the degree of implementation of the HS program. There can be a large variety of implementation in schools with the HS program certificate, but schools without HS certification might also have adopted either SHP in general or specifically the HS program. Future studies should explore these unaddressed factors. Another limitation was the use of data from different PHSs, which led to variations in whether children were measured with or without clothing. Although adjustments were made for clothing, future research should adhere to a standardized protocol for all children, to ensure greater accuracy and consistency. The YHC also does not have data on all children, primarily due to non-response [61], which may have introduced selection bias. Additionally, only three PHSs registered the total SDQ score (and incidentally, the subscale scores), while it was unknown how many questions were filled out. Therefore, the SDQ data of these PHSs could not be included. Moreover, the HS register is adjusted if a school merges or splits, leading to potential inaccuracies in classifying all schools correctly [62].

## 5. Conclusions

School differences explained a small part of the variation between primary school students regarding the BMI z-score and psychosocial health, and these differences were mostly explained by SES. Our results indicated that HS certification had a small, but favorable association with the BMI z-score of students, but not their psychosocial health. Accordingly, obtaining HS certification might contribute to the better physical health of primary school students in general. This might indicate that HS certification also relates to healthier lifestyles in primary schools, but further research should examine this.

## Figures and Tables

**Table 1:** Descriptive statistics of sample of primary schools separately for (subsample of) certified schools and non-certified schools.

	BMI z-Score (N = 7077 ^2^)			SDQ Score (N = 2826 ^2^)		
	Certified Schools ^1^ (N = 1415 ^2^)	Non-Certified Schools ^1^ (N = 5662 ^2^)	Subsample Certified Schools ^1^ (N = 951 ^2^)	Certified Schools ^1^ (N = 750 ^2^)	Non-Certified Schools ^1^ (N = 2076 ^2^)	Subsample Certified Schools ^1^ (N = 476 ^2^)
Schools (N)	332	1366	210	202	639	106
Students (N)	52,650	226,128	33,645	32,373	94,966	19,679
BMI z-score (Mean (SD))/SDQ score ^3^ (Mean (SD))	−0.14 (1.02)	−0.18 (0.98) *	−0.15 (1.00)	5.80 (4.70)	5.57 (4.60) *	5.89 (4.73)
**School health promotion**						
No. of years Healthy School (Mean (SD))	1.76 (1.67)	- *	1.08 (1.23) *	1.93 (1.76)	- *	1.04 (1.21) *
*Healthy School topic certificates (yes) (%)*						
Nutrition	26.50	- *	23.97	30.53	- *	25.00 *
Physical activity	27.92	- *	20.19 *	28.80	- *	21.22 *
Well-being	25.44	- *	18.19 *	24.67	- *	15.55 *
Relationships and sexuality	3.25	- *	2.00	3.73	- *	2.94
Environment and nature	1.34	- *	^4^	^4^	-	^4^
**General school characteristics**						
Urbanicity school area (%)						
High	30.88	28.24	25.55 *	37.60	39.93	36.55
Medium	19.65	20.22	21.35	18.40	17.34	23.53 *
Low	49.47	51.54	53.10	44.00	42.73	39.92
School size (no. of students) (Mean (SD))	214 (116)	206 (116) *	207 (110)	217 (125)	221 (135)	207 (108)
School type (%)						
Public	28.27	25.42 *	25.24	29.07	25.39	26.05
Independent non-denominational	3.75	3.30	^4^	^4^	5.30	^4^
Catholic	43.18	36.28 *	44.69	52.67	41.52 *	60.50 *
Protestant	23.53	31.91 *	24.40	13.47	24.86 *	9.03 *
Other	1.27	3.09 *	^4^	^4^	2.94	^4^
**School population characteristics (Mean (SD))**						
Age in months	101.19 (17.78)	97.77 (16.87) *	101.86 (18.16)	99.41 (13.54)	99.50 (12.48)	99.99 (13.80)
Gender (proportion boy)	0.50 (0.11)	0.50 (0.11) *	0.50 (0.11)	0.49 (0.10)	0.50 (0.10) *	0.49 (0.10)
Proportion disadvantaged students	0.11 (0.11)	0.08 (0.09) *	0.10 (0.11) *	0.13 (0.12)	0.08 (0.10) *	0.13 (0.12)
Proportion high parental educational attainment ^5^	0.54 (0.20)	0.60 (0.18) *	0.55 (0.20)	0.53 (0.22)	0.61 (0.19) *	0.51 (0.22)
Proportion high household income ^5,6^	0.47 (0.20)	0.54 (0.18) *	0.48 (0.19)	0.45 (0.21)	0.55 (0.19) *	0.44 (0.22)
Proportion low household income ^5,6^	0.03 (0.05)	0.04 (0.05) *	0.04 (0.05)	0.04 (0.06)	0.03 (0.05) *	0.05 (0.06)
Proportion first-generation migration background ^5,6^	0.04 (0.06)	0.03 (0.06) *	0.03 (0.06)	0.05 (0.07)	0.04 (0.06) *	0.04 (0.07)
Proportion second-generation migration background ^5,6^	0.17 (0.18)	0.13 (0.15) *	0.15 (0.17) *	0.20 (0.19)	0.15 (0.14) *	0.19 (0.18)
Proportion high urbanicity (home) area	0.30 (0.41)	0.26 (0.39) *	0.25 (0.39) *	0.38 (0.43)	0.37 (0.43)	0.38 (0.42)
Proportion low urbanicity (home) area	0.51 (0.46)	0.56 (0.46) *	0.54 (0.46)	0.44 (0.46)	0.48 (0.46) *	0.41 (0.46)
Proportion overweight and underweight ^7^	-	-	-	0.19 (0.11)	0.17 (0.11) *	0.20 (0.12)

Note: - = not applicable. * significantly different compared to certified schools (*p* < 0.05). ^1^ Certified schools have had the HS program certificate at least once since the beginning of the program (2010), non-certified schools have never obtained the HS program certificate within the study period, and the subsample of certified schools obtained the HS program certificate within our study period. ^2^ Unless otherwise stated, this refers to the total count of school × school year combinations with available data. Results are summarized per school, separately for each school year. ^3^ Results for outcomes are displayed at the individual level, instead of the school year level. For the BMI z-score, there were no missing values. For the SDQ score, there were missing values of 5844 students in certified schools, 15,778 students in non-certified schools, and 2687 students in the subsample of certified schools. ^4^ Descriptive statistics (including * for significant differences) are not reported due to privacy reasons. ^5^ For the SDQ score: For certified schools, data were available from 739 school × school year combinations. For non-certified schools, data were available from 2020 school × school year combinations. For the subsample of certified schools, data were available from 472 school × school year combinations. ^6^ The proportion of students with a medium household income was used as a reference. The proportion of native students was used as a reference. The proportion of students living in a medium urbanicity area was used as a reference. ^7^ For the SDQ score: For certified schools, data were available from 690 school × school year combinations. For non-certified schools, data were available from 1915 school × school year combinations. For the subsample of certified schools, data were available of 433 school × school year combinations. No = number; SD = standard deviation.

**Table 2:** Multilevel intraclass correlations in primary schools for BMI z-scores and SDQ scores.

	BMI z-Score (N = 7077 ^1^)		SDQ Score (N = 2826 ^1^)	
	ICC School-Level (%)	ICC School Year Level (%)	ICC School-Level (%)	ICC School Year Level (%)
0 model	2.41	0.62	2.45	0.45
**School health promotion**				
Healthy School	2.40	0.62	2.43	0.45
Healthy School ever	2.38	0.62	2.40	0.45
Number of years Healthy School	2.40	0.62	2.43	0.45
*Healthy School topic certificates*				
Nutrition	2.41	0.62	2.43	0.45
Physical activity	2.38	0.63	2.42	0.45
Well-being	2.41	0.62	2.45	0.45
Relationships and sexuality	2.41	0.62	2.45	0.45
Environment and nature	2.41	0.62	- ^2^	- ^2^
**General school characteristics**				
School size	2.28	0.63	2.31	0.46
School type	2.36	0.62	2.39	0.45
Urbanicity school area ^3^	2.40	0.62	2.42	0.45
**School population characteristics**				
Disadvantaged students	1.18 *	0.65	1.49 *	0.45
High parental educational attainment ^3^	0.90 *	0.77	1.18 *	0.58
Household income ^3^	1.42 *	0.72	1.42 *	0.56
Migration background ^3^	1.77 *	0.68	2.03 *	0.47
Urbanicity home area ^3,4^	2.33	0.63	2.36	0.45
Overweight and underweight ^4^	-	-	1.96 *	0.50
All significant variables multivariately ^5^	0.83	-	1.12	-

Note: All analyses were adjusted for the standardized age of the students and for the Public Health Service. * Inclusion of the variable resulted in a decrease of at least 10% in the ICC. ^1^ Refers to the number of school year × school combinations included in the analyses. ^2^ Results are not presented since less than 10 schools had the topic certificate. ^3^ Consists of two variables. ^4^ The analysis was adjusted for the characteristic on the individual level. ^5^ Variables were only included in the multivariate analysis if their inclusion resulted in a decrease of at least 10% in the ICC. Results were not provided for the school year level, since the ICC did not decrease by ≥10% after the inclusion of the variables. N (BMI z-score): students = 278,778; schools = 1698. N (SDQ): students = 127,339; schools = 841. - Indicates that the analysis was not conducted.

**Table 3:** Association between Healthy School certification and BMI z-scores and SDQ scores of students in primary schools.

	BMI z-Score (N = 951 ^1^)		SDQ Score (N = 476 ^1^)	
	B	95% CI	B	95% CI
Model 1: Intercept	−0.03	(−0.14, 0.08)	6.89	(6.16, 7.62) *
HS	−0.03	(−0.06, −0.01) *	−0.06	(−0.23, 0.11)
Model 2: Intercept	−0.04	(−0.15, 0.07)	6.88	(6.15, 7.61) *
Number of years HS	−0.01	(−0.02, 0.00)	−0.01	(−0.09, 0.06)
Model 3: Intercept ^2^	−0.07	(−0.19, 0.04)	-	-
No HS	0.04	(0.01, 0.07) *	-	-
HS, but no nutrition/physical activity certificate	0.04	(−0.01, 0.08)	-	-
Model 4: Intercept ^3^	-	-	6.60	(5.82, 7.39) *
No HS	-	-	0.25	(−0.02, 0.52)
HS, but no well-being certificate	-	-	0.27	(−0.02, 0.56)

Note: Only schools are included that became a certified school in our dataset. All analyses have been adjusted for the Public Health Service, the standardized age of the students, and characteristics that explained ≥10% differences between schools in Table 2. Parameters are not presented for these control variables. Association was not tested since only the association with the most relevant topic certificate(s) with regard to the outcomes were tested. ^1^ Total count of school × school year combinations. For the BMI-z score, 210 schools and 33,645 students were included in our analyses. For the SDQ score, 106 schools and 19,679 students were included in our analyses. ^2^ Having the nutrition or physical activity certificate is used as a reference group. ^3^ Having the well-being certificate is used as a reference group. * = *p*-value < 0.05. HS = Healthy School program certificate. - Indicates that the analysis was not conducted.

**Table 4:** Interaction between school population characteristics and HS certification on the BMI z-scores and SDQ score.

	BMI z-Score (N = 951 ^1^)		SDQ Score (N = 476 ^1^)	
	B	95% CI	B	95% CI
Model 1:				
Intercept	−0.03	(−0.14, 0.09)	6.82	(6.09, 7.56) *
Disadvantaged students	0.67	(0.36, 0.99) *	3.10	(1.12, 5.07) *
HS	−0.06	(−0.10, −0.03) *	0.08	(−0.17; 0.32)
HS × disadvantaged students	0.31	(0.05, 0.58) *	−1.20	(−2.83; 0.44)
Model 2:				
Intercept	−0.07	(−0.19, 0.06)	7.03	(6.25, 7.80) *
High parental educational attainment	−0.18	(−0.35, −0.01) *	−0.46	(−1.56, 0.64)
HS	0.02	(−0.06, 0.11)	−0.31	(−0.80, 0.19)
HS × high parental educational attainment	−0.10	(−0.24, 0.04)	0.46	(−0.40, 1.31)
Model 3:				
Intercept	−0.08	(−0.20, 0.05)	7.12	(6.31, 7.93) *
Low household income	0.09	(−0.42, 0.59)	−2.29	(−5.20, 0.62)
High household income	0.10	(−0.06, 0.25)	−1.64	(−2.73, −0.56) *
HS	0.05	(−0.05, 0.14)	−0.47	(−1.10, 0.15)
HS × low household income	0.02	(−0.64, 0.67)	1.82	(−2.18, 5.82)
HS × high household income	−0.16	(−0.32, 0.00) *	0.72	(−0.32, 1.77)
Model 4:				
Intercept	−0.03	(−0.15, 0.08)	6.82	(6.07, 7.57) *
First-generation migration background	0.30	(−0.16, 0.76)	0.64	(−1.89, 3.17)
Second-generation migration background	−0.16	(−0.31, 0.02)	0.41	(−0.62, 1.44)
HS	−0.04	(−0.08, −0.01) *	0.09	(−0.18, 0.37)
HS × first-generation migration background	−0.44	(−0.99, 0.11)	−0.44	(−3.44, 2.57)
HS × second-generation migration background	0.17	(0.01, 0.33) *	−0.75	(−1.96, 0.47)
Model 5:				
Intercept	-	-	6.92	(6.15, 7.69) *
Overweight and underweight	-	-	0.24	(−1.73, 2.20)
HS	-	-	−0.10	(−0.55, 0.34)
HS × overweight and underweight	-	-	0.25	(−2.09, 2.59)

Note: Only schools are included that became a certified school in our dataset. All analyses have been adjusted for the Public Health Service, the standardized age of the students, and characteristics that explained ≥10% differences between schools in Table 2. For the SDQ score, analyses were also adjusted for the individual BMI z-score of the students. Parameters are not presented for these control variables. Except for HS certification, all presented variables are included in the analyses as continuous variables, i.e., proportion. ^1^ Total count of school × school year combinations. For the BMI z-score, 210 schools and 33,645 students were included in our analyses. For the SDQ score, 106 schools and 19,679 students were included in our analyses. HS = the Healthy School program certificate. * = *p*-value < 0.05. - Indicates that the analysis was not conducted.

## Data Availability

Restrictions apply to the availability of these data. Data were obtained from seven regional Public Health Services (GGD’en in Dutch), the Netherlands Initiative for Education Research (NRO) at Statistics Netherlands (CBS), and the Healthy School organization. Data are available with the permission of the GGD’en, NRO, CBS, and the Healthy School organization.

## Supplementary Materials

The following supporting information can be downloaded at: https://www.mdpi.com/article/10.3390/ijerph21081073/s1, Figure S1: Flowchart primary school students.
