# Supplementary material for: School Health Promotion, the Body Mass Index z-Score, and Psychosocial Health in Primary Schools of the Netherlands
Source: Int J Environ Res Public Health. 2024 Aug 15;21(8):1073. doi: 10.3390/ijerph21081073 (PMC11353774; doi:10.3390/ijerph21081073)
Supplement: Supplementary file 1 [file ijerph-21-01073-s001.zip › ijerph-3116964-supplementary.pdf]

Supplementary Materials

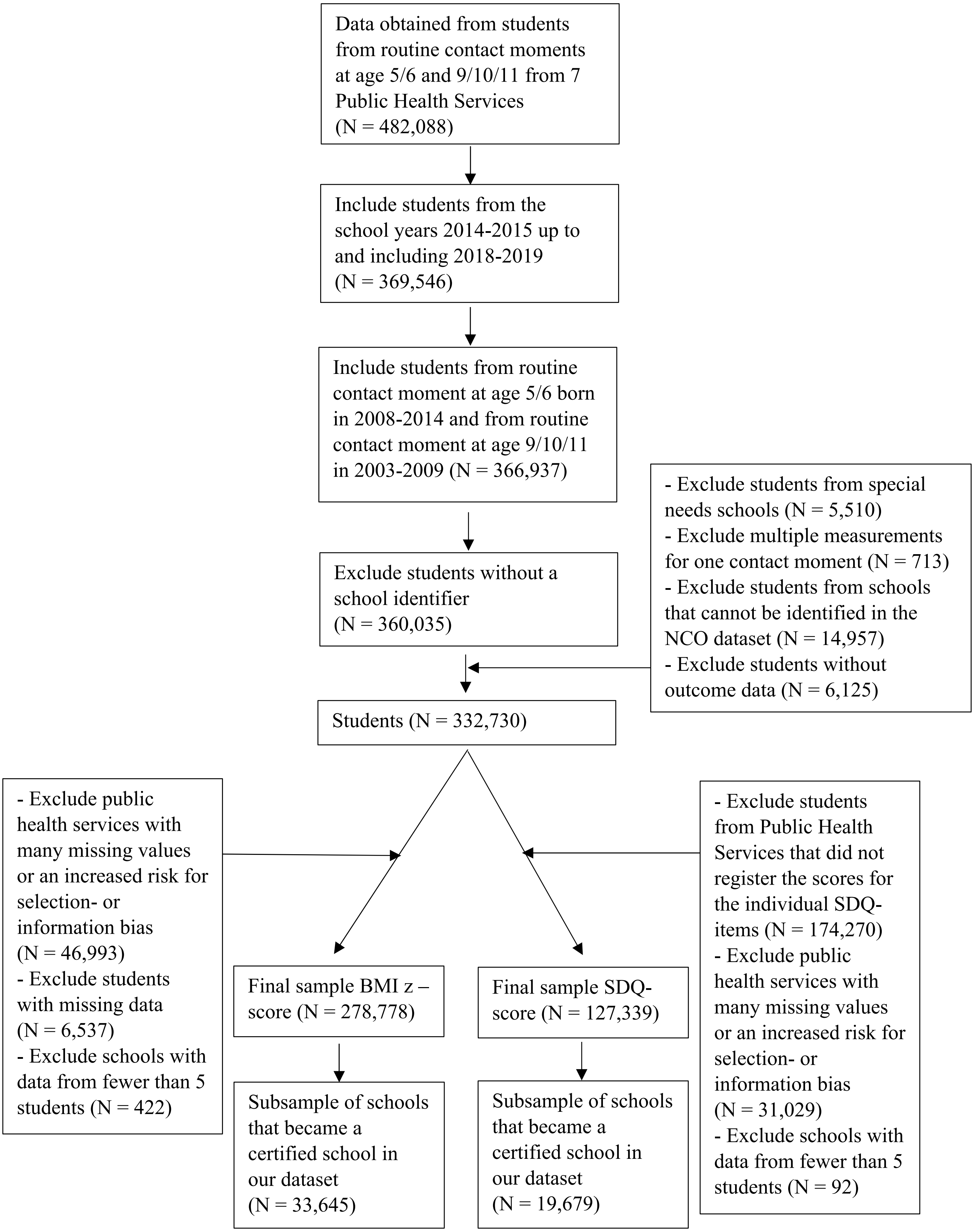

Figure S1: Flowchart primary school students
Note: N refers to students
